# Exploration of extraterrestrial biosignatures and planetary habitability: A prospect

**DOI:** 10.1016/j.fmre.2026.07.006

**Published:** 2026-07-29

**Authors:** Zengqian Hou, Jizhong Liu, Yuming Wang, Lei Geng, Hui Huang, Hailiang Dong, Yazhou Dong, Siteng Fan, Jihua Hao, Jiansen He, Dali Kong, Hui Li, Yan Li, Yiliang Li, Mang Lin, Zongcheng Ling, Yuangao Qu, Zhenfeng Sheng, Tong Wang, Yuxian Wang, Bo Xu, Jun Yang, Bingkun Yu, Xiao Zhang

**Affiliations:** a Institute of Deep Space Sciences, Deep Space Exploration Laboratory, Hefei 230088, China; b Institute of Geology, Chinese Academy of Geological Sciences, Beijing 100037, China; c Lunar Exploration and Space Engineering Center, China National Space Administration, Beijing 100037, China; d School of Earth and Space Sciences, University of Science and Technology of China, Hefei 230026, China; e State Key Laboratory of Geomicrobiology and Environmental Changes, China University of Geosciences, Beijing 100083, China; f Department of Earth and Space Sciences, Southern University of Science and Technology, Shenzhen 518055, China; g School of Earth and Space Sciences, Peking University, Beijing 100871, China; h Shanghai Astronomical Observatory, Chinese Academy of Sciences, Shanghai 200030, China; i National Space Science Center, Chinese Academy of Sciences, Beijing 100190, China; j Department of Earth & Planetary Sciences, The University of Hong Kong, Hong Kong 999077, China; k Guangzhou Institute of Geochemistry, Chinese Academy of Sciences, Guangzhou 510640, China; l Shandong Key Laboratory of Optical Astronomy and Solar-Terrestrial Environment, School of Space Science and Technology, Institute of Space Sciences, Shandong University, Weihai 264209, China; m Institute of Deep-Sea Science and Engineering, Chinese Academy of Sciences, Sanya 572000, China; n Institute of Overall Design, China Academy of Space Technology (CAST), Beijing 100094, China; o National Mineral Rock and Fossil Specimens Resource Center, and Deep Space Exploration Laboratory, China University of Geosciences, Beijing 100083, China; p Laboratory for Climate and Ocean-Atmosphere Studies, Department of Atmospheric and Oceanic Sciences, School of Physics, Peking University, Beijing 100871, China

**Keywords:** Extraterrestrial biosignatures, Habitable environment, Mars, Venus, Icy satellites

## Abstract

China’s deep space exploration has achieved remarkable accomplishments and is now undergoing a significant strategic transition from an engineering-driven program to one that integrates engineering and science. This shift provides a timely opportunity for China to systematically define the development directions and overall framework of planetary science by targeting scientific frontiers, focusing on key research areas, and addressing major scientific questions. In this context, we provide an in-depth analysis of the crucial role of searching for potential biosignatures and investigating planetary habitability for understanding the origin of life. We systematically review the current research state and emerging trends in this field at both the domestic and international levels. Furthermore, we identify three key scientific themes that require urgent attention: planetary habitability, the search for extraterrestrial biosignatures, and the co-evolution of life and habitable environments. For each theme, the major underlying scientific questions are further clarified. Based on these scientific priorities, we also propose key target celestial bodies and critical detection scenarios for future missions. The results are expected to provide important theoretical support for defining scientific objectives and informing the top-level design in China’s future planetary exploration endeavors.

## 1 Introduction

Is life on Earth unique, and are we alone in the universe? These fundamental scientific and philosophical questions have intrigued humanity for millennia. The origin of life remains a major frontier in science and, after years of vigorous development, is now at a critical juncture transitioning from incremental progress to groundbreaking advances. Systematic research into detecting of extraterrestrial biosignatures and assessing planetary habitability holds promise for generating highly influential scientific discoveries, improving our understanding of Earth’s formation and evolution, and enabling better predictions of its future.

Based on extensive prior research, several origin-of-life hypotheses have been proposed, such as the submarine hydrothermal vent origin [1] and panspermia life seeds [2]. However, intense geological activities on Earth have obscured key lines of evidence, complicating efforts to study the origin and early evolution of life on our planet. Planetary exploration offers a promising alternative: the relatively well-preserved surfaces of other celestial bodies and the potential existence of habitable environments create valuable opportunities to search for potential extraterrestrial biosignatures and to investigate processes relevant to life’s origin and evolution. Moreover, by studying multi-sphere interaction, including planetary atmospheres, stratigraphy, and magnetic fields, based on which we may clarify the mechanisms of life’s origin and establish relationships between life and critical environmental factors.

Key planetary conditions such as liquid water, magnetic fields, suitable temperature ranges, and appropriate atmospheric composition are fundamental for forming habitable environments and are essential for the origin of life [3]. Research indicates that if a planet’s early surface environment was weakly oxidizing and dominated by CO_2_ and N_2_ [4,5], the synthesis and accumulation of organic molecules would have been significantly inhibited. Although highly reducing submarine hydrothermal systems have been proposed as potential cradles of life [6,7], they face challenges such as low yields of organic synthesis and difficulties in preserving products over geologically meaningful timescales. In recent years, water-rock reactions [8,9] and widespread early meteorite impacts [10] have been shown to generate reduced gases such as H_2_, CH_4_, and NH_3_, offering new potential pathways for the development of planetary habitability and the origin of life [11].

Even if a planet becomes habitable, how a prebiotic world transitions into a biotic one remains unknown. After the formation of monomeric organic molecules, widely distributed minerals on planetary surfaces can provide catalytic sites and protective environments for the further generation, enrichment, and polymerization of organic compounds [[12], [13], [14], [15]], thereby facilitating the formation of organic macromolecules. Some minerals may also exhibit enzyme-like activity [16,17], potentially catalyzing the polymerization of organic macromolecules into biomolecules and triggering the origin of life. However, the mechanisms by which mineral surfaces and enzyme-like catalysts drive energy metabolism and material exchange remain largely unresolved.

After the emergence of life, it may in turn modify its environment through metabolic activities [18], while changes in planetary habitability can exert strong feedback on the evolution of the biosphere [19]. Through such continuous environment-life interactions, both habitability and the biosphere have undergone significant co-evolution. Evidence preserved on the ancient surfaces/subsurfaces of celestial bodies such as Mars, Venus, and icy satellites therefore offers crucial windows into the processes relevant to life’s origin, while the evolutionary histories of their atmospheres, magnetic fields, and mineral species provide important constraints for understanding the formation, maintenance, and transformation of habitable environments.

Although the global scientific community has made significant progress on major questions, such as how environmental habitability formed, how life originated in these environments, and how life and habitability co-evolved, definitive answers remain elusive [20]. Addressing these issues requires systematic research on extraterrestrial biosignature detection and planetary habitability, including reconstructing the evolutionary history of planetary habitability, exploring the transition from a prebiotic world to a living one, inferring possible evidence of life from environmental features, identifying conditions where life may exist, and studying the relationship between habitability and life. Conducting deep space exploration missions to search for habitable celestial bodies and potential extraterrestrial biosignatures is the fundamental approach to answering the profound scientific and philosophical question of whether life on Earth is unique and whether we are alone in the universe.

This review is organized into several sections. Section 2 summarizes recent progress in the exploration of potential extraterrestrial biosignatures and planetary habitability research. Section 3 outlines the core scientific themes and fundamental questions currently defining this field. Building on these themes, Section 4 identifies potential target celestial bodies and promising research scenarios. Section 5 discusses China’s current research capabilities and the development roadmap for future deep space exploration. The review concludes with a summary and outlook in Section 6.

## 2 Research status and development trends

### 2.1 Scientific research progress

The search for extraterrestrial biosignatures is the central scientific endeavor to understand the humanity’s place in the universe, making it the strategic priority in deep space exploration. Current research focuses on three main areas: the evolution of habitable environments, the search for biosignatures, and the mechanisms of life’s origin, forming a multi-scale exploration chain from planetary environments to biosignatures. This section systematically reviews scientific research progress through three progressive dimensions: “the Solar System habitable zones”, “traces of water”, and “origin of life”.

#### 2.1.1 The solar system habitable zones (Mars and Venus)

Mars and Venus, as the rocky planets similar to Earth in the Solar System, provide critical references for understanding planetary environmental evolution and the potential for life.

Mars, owing to its geological similarity to Earth, has long been a primary target in the search for extraterrestrial habitability and life. The existence of an ancient ocean on Mars is a central focus of habitability research. Early Viking orbiter images led Lucchitta et al. to propose the first evidence of marine sediments in the northern hemisphere [21]. Parker et al. further delineated two putative paleoshorelines: the larger Noachian Arabia shoreline and the smaller Hesperian Deuteronilus shoreline [22]. The Vastitas Borealis Formation (VBF), widely distributed across the northern plains and interpreted as marine sediments associated with outflow channel floods [23], coincides with the Deuteronilus shoreline; crater counting dates the VBF to approximately 3.6 billion years ago, suggesting an ocean at that time [24]. The ground-penetrating radar data from Zhurong rover revealed 76 layers of inclined sedimentary structures whose dielectric constant matches that of coastal sand and gravel on Earth, providing the first radar confirmation of a long-term, stable shallow-sea environment at mid-to-low latitudes on Mars approximately 3.6 billion years ago [25]. This finding extends the period of liquid water activity on Mars to hundreds of millions of years and expands the habitable zone from high latitudes to the more temperate and topographically flat mid-to-low latitudes, offering a “prime location” for selecting a future Mars base.

Recent work by Hou et al. provides a major mineralogical advance in reconstructing the Hesperian ocean in Utopia Planitia [26]. By identifying manganese (hydr)oxides as a geochemical recorder of aqueous evolution, and combining a dedicated spectral dataset with quantitative detection methods, the study moves evidence for the northern Martian ocean beyond geomorphological interpretation to direct mineralogical constraint. The results indicate that this ocean was not a short-lived water body, but a dynamically evolving system that persisted for at least million-year timescales, implying sustained redox-active aqueous environments with important implications for habitability.

In addition to paleo-ocean evidence, Martian lava tubes offer a new perspective for habitability assessment. The present Martian surface is extremely hostile to life: a thin atmosphere allows intense ultraviolet radiation, the mean annual temperature is as low as 215 K, and the soil is considered strongly oxidizing [27]. Consequently, the subsurface may be more habitable, and lava tubes serve as natural portals to this underground realm. Numerous lava tubes have been identified in volcanic regions such as Arsia and Olympus Mons. Their tens-of-meters-thick basalt roofs can effectively shield against solar radiation, micrometeorites, and drastic temperature fluctuations, while the internal temperature is believed to be constant [28]. Such stable thermal conditions favor the presence of liquid water in the form of ice or transient liquids, and volcanic minerals on tube walls can provide nutrients for chemosynthetic microorganisms [27]. Furthermore, secondary sulfate minerals within lava tubes have been shown to trap and preserve organic molecules (e.g., lipids, alkanes) over long periods, making them ideal carriers of potential biosignatures [29]. Thus, Martian lava tubes are not only natural shelters for future crewed missions but also prime targets for searching for extant or extinct life, and their stable, potentially water- and energy-rich subsurface environment further enriches the picture of Martian habitability.

Venus has become a frontier in astrobiology because its evolution indicates a possible water-rich past and its present-day cloud layer provides a temperate, chemically active environment that may host prebiotic chemistry or even microbial life [30]. The D/H ratio in the Venusian atmosphere is 100 times that of Earth and can increase to 1500 times with altitude [31], indicating substantial hydrogen escape during Venus’s evolution and providing strong evidence that large amounts of liquid water may have existed on the early Venusian surface [32,33]. Venus formed similarly to Earth, and water vapor could have condensed to form a liquid ocean as the planet cooled [34]. However, Venus orbits at 0.72 AU and receives nearly twice the solar flux of Earth. Whether early liquid water could have been sustained remains debated. One view holds that the extremely slow rotation allows water evaporated at the subsolar point to form high-altitude clouds that reflect solar radiation, suppressing warming and maintaining liquid water [35,36]. An opposing view argues that the early Venusian surface was enveloped by hot H_2_O gas, preventing cloud formation on the dayside; clouds form instead on the nightside and absorb outgoing longwave radiation, inhibiting cooling and preventing the formation of a liquid ocean [37]. Regardless, cloud feedback plays a critical role in Venus’s habitability evolution.

Today, although the surface of Venus is inhospitable, the cloud layer at 50–65 km altitude experiences temperate conditions (300–350 K, 0.1–1 atm) [38], making it a potential habitat. Remote sensing has revealed strong ultraviolet absorbers in the Venusian clouds, whose identity remains unknown. Some studies propose that biochemical components of microorganisms (e.g., ferritin, photosynthetic pigments) could contribute to this absorption [39,40]; others suggest that organic molecules possibly derived from meteorites, photochemistry, or even biological activity are the main absorbers [41]. Moreover, the reported detection of 10 ppb phosphine (PH_3_) in Venus’s clouds [42] attracted considerable attention. That study, after ruling out atmospheric chemistry and known abiotic production pathways, suggested that PH_3_ might originate from unknown chemical or geological processes, or even biological activity. However, this interpretation remains highly controversial. Subsequent analyses have attributed the reported signal to data reduction artifacts, interference from SO_2_, or revising the upper limit to only a few ppb or lower [43,44]. More broadly, recent studies of other planetary and substellar atmospheres have shown that phosphorus partitioning can be strongly influenced by mineral and condensate chemistry [45]; for example, phosphine depletion in brown dwarf atmospheres has been interpreted as the result of metal phosphide formation. Although such mechanisms cannot be directly transferred to Venus, they underscore the broader point that PH_3_ abundance is highly sensitive to complex non-biological chemistry, and therefore caution is warranted when interpreting phosphine as a standalone biosignature.

Recent experimental work has also broadened the chemical context of Venusian habitability. In a Venus-like atmosphere, photoactive elemental sulfur allotropes have been shown to promote extensive ammonia synthesis, revealing a previously underappreciated abiotic pathway for reduced nitrogen production [46]. This result is potentially important for astrobiology because ammonia is a key feedstock for prebiotic chemistry and may also help buffer the extreme acidity of Venusian cloud droplets, thereby improving the prospects for chemical complexity or even microbial survival in the cloud layer. Together with earlier findings that simple organic precursor molecules can be transformed into more complex organics in concentrated sulfuric acid [47], these results suggest that the Venusian atmosphere may support a richer inventory of reactive and potentially prebiotically relevant species than previously assumed.

Experiments on the concentrated sulfuric acid environment of the Venusian clouds have shown that simple organic precursor molecules can transform into complex organic compounds [41], and nucleic acid bases [48] as well as 19 out of 20 tested amino acids [47] exhibit remarkable stability in concentrated sulfuric acid. Seager et al. also proposed that microorganisms could alternate between desiccated spores and active states to adapt to cloud cycling [38]. These findings challenge the traditional view that life must depend on water and provide chemical support for sulfuric acid droplets as a potential medium for non-aqueous life.

Although both Mars and Venus are currently inhospitable at their surfaces, they offer complementary evidence for planetary habitability. Mars preserves evidence for an ancient ocean, chemically dynamic aqueous environments, and potentially habitable subsurface lava-tube systems, whereas Venus offers a temperate cloud layer, active sulfur photochemistry, and the possibility of non-aqueous chemical pathways relevant to habitability. The evidence on Mars points to long-term liquid water and subsurface refugia, while the Venusian case emphasizes cloud-layer chemistry, organic stability in concentrated sulfuric acid, and alternative chemical routes that may sustain prebiotic or even biological processes. Together, they broaden our understanding of the boundaries of planetary habitability.

#### 2.1.2 Traces of water (Mars and icy satellites)

As water is essential to life on Earth, its distribution and forms on other celestial bodies serve as core clues in the search for life. Previous studies have advanced along two paths: “traces of water on Mars” and “subsurface water on icy satellites”, forming a multi-scale, technologically synergistic detection framework with key findings across multiple bodies.

The distribution of water on Mars spans a vast range, from the surface to the subsurface and from the polar regions to mid-to-low latitudes. High-resolution imaging and spectral data have revealed extensive exposures of water ice in mid-latitude scarps [49]. Orbital radar sounding has uncovered polar layered ice deposits and shallow subsurface ice at mid-to-high latitudes [50,51], indicating that water ice is stored in multiple layers and at various depths. Xu et al. detected ice-rich layers over a hundred meters thick with a water ice volume fraction of 55%–85% in central Utopia Planitia, with the possibility of even thicker ice bodies beneath [52]. Zhurong rover’s radar discovered extensive ice-rich layers at depths of 10–35 m [53] and identified buried paleopolygonal terrain [54], reflecting environmental evolution involving freezing, thawing, or ice-bearing sediments. The Martian atmospheric water cycle is also active: during dust seasons, water vapor can be transported up to an altitude of 100 km and become supersaturated, enhancing water escape [55]. Hydrous minerals provide direct evidence of ancient water–rock interactions: orbital hyperspectral data have identified phyllosilicates, carbonates, and opal [56]; the Curiosity rover revealed a transition from lacustrine to arid evaporative environments [57]; the Perseverance rover discovered neutral-pH liquid water environments persisting for over 1000 years [58]; and the Zhurong rover found hydrated sulfates [59], suggesting episodic water activity. Geomorphological features of liquid water, such as valley networks, deltas, and sand dune crust cracks [60], further corroborate the history of water activity. Recently, a deep-learning-based Spectral Contrastive-Aware Network designed to decode the short-wave infrared spectral data from China’s Zhurong rover and ESA/NASA orbiters has revealed an altitude-dependent enrichment of manganese (hydr)oxides in Utopia Planitia, forming a mineralogical “bathtub ring” that delineates the boundaries of an ancient Hesperian ocean. The reconstructed ocean persisted for 0.8–1.5 million years, providing quantitative evidence of prolonged surface water stability on early Mars [26]. From deeply buried ice-rich layers to recent brine activity, evidence for water on Mars spans different spatial scales and geological epochs, demonstrating that the planet once possessed habitable conditions conducive to the origin of life.

Multiple icy satellites are suggested to have stable subsurface liquid water oceans or pockets. These water reservoirs are usually sandwiched by thick icy shells and typically harbor a rocky or metallic core. The maintenance of liquid water activities over long geological timescales is powered by endogenic energy sources, including tidal heating and radioactive decay, which may potentially drive chemosynthetic ecosystems that provide energy and chemical nutrients for life [61]. To date, compelling evidence indicates that Enceladus, Europa, Ganymede, and Callisto possess subsurface oceans; Titan, Triton, Pluto, and others are considered potential “ocean worlds” [62]. The coexistence of an icy shell and subsurface liquid water on icy satellites, combined with chemical environments driven by stable heat sources, forms the core basis for their habitability.

A synergistic investigation of Martian and icy-satellite water systems reveals that water distribution across the Solar System is neither spatially isolated nor temporally independent.

#### 2.1.3 Origin of life (small solar system bodies and icy satellites)

Understanding the chemical evolution path toward life is an ultimate goal of astrobiology. Previous studies employed a dual strategy—sample return from small bodies and in-situ icy satellite exploration—to trace the distribution and evolution of prebiotic materials in the Solar System.

Small bodies (asteroids and comets) preserve pristine material from the solar nebula 4.6 billion years ago and are key to understanding the origin of life’s precursor compounds. They serve as “time capsules” for revealing the sources of key ingredients for the origin of life on Earth. Multiple exploration missions, through sample return and in situ analyses, have provided direct evidence for the “exogenous delivery” hypothesis—those comets or asteroids delivered water and prebiotic organic molecules to the early Earth. The Stardust mission was the first to return dust samples from comet 81P/Wild2 [63]. Analyses revealed the presence of amino acids (e.g., glycine) and complex organic compounds containing carbon, hydrogen, oxygen, and nitrogen [64], confirming that comets could indeed have delivered essential precursor substances for life to Earth. Japan’s Hayabusa2 mission returned 5.4 g of samples from Ryugu, marking the first extraterrestrial confirmation of >20 amino acids, along with liquid water and approximately 20,000 organic compounds [65]. The US OSIRIS-REx mission returned 121.6 g of samples from the near-Earth asteroid Bennu; preliminary analyses indicate the presence of water (in the form of hydroxyl groups within clay minerals) and carbon in abundance as high as ∼5 wt.% [66]. Together, these two missions strongly support the hypothesis that asteroids delivered water and prebiotic precursors to Earth.

China’s Tianwen-2 mission employs an “dual-target exploration and layered sampling” approach [67,68]. For the near-Earth asteroid 2016 HO_3_—whose orbital resonance suggests it may be an impact ejecta from the early Moon [[69], [70], [71]]—100 g of stratified samples will be collected [68]. High-precision isotope analyses could test the hypothesis of early material exchange between Earth and the Moon. For the main-belt comet 311P, the mission will conduct fly-by observations to capture polycyclic aromatic hydrocarbons and amino acid precursors (e.g., glyoxylic acid) in its outgassing plumes.

Compared to Japan’s Hayabusa2 and NASA’s OSIRIS-REx missions, Tianwen-2 will conduct coordinated observations of a dynamic comet and a static asteroid, establishing a comprehensive research framework combining sample return and in-situ monitoring. Its payload includes a diffraction rotational snapshot spectral camera [72] and a Charged and Neutral Particle Analyzer [73] to study solar wind modification of organic compounds on small bodies, providing empirical data on organic stability under space weathering.

Icy satellites in the outer Solar System are distinguished by a thick ice shell and a global or regional subsurface liquid water ocean [74]. Enceladus provides the most compelling evidence for such an ocean world. The Cassini-Huygens mission detected active south polar plumes, indicating direct material exchange between the subsurface ocean and the surface [75,76]. The plume contains water vapor, ice grains, salts, ammonia, organic compounds, and phosphates, confirming a global high-salinity ocean possessing nearly all essential ingredients for life [[77], [78], [79]]. To assess its habitability and search for biosignatures, a flagship Orbilander mission concept has been proposed [80,81]. Europa also exhibits strong evidence for a global ocean, including complex tectonics [82], an induced magnetic field [83], and potential plume activity [84,85]. NASA’s ongoing Europa Clipper mission [86] and ESA’s JUICE mission [87] are specifically designed to assess Europa’s habitability.

Triton, a Kuiper Belt object and ocean world candidate, displays a young surface and nitrogen-driven cryovolcanic plumes, suggesting internal heat and a possible subsurface ocean [88,89]. Its origin-of-life potential extends beyond hydrothermal energy: atmospheric photochemistry produces organic haze containing oxygen-rich molecules, including glyceraldehyde and all five biological nucleobases, which settle onto the surface [90]. Impact events could deliver these precursors to the subsurface ocean, where catalytic reactions on mineral surfaces generate key biomolecules such as threonine, aspartic acid, and xanthine [91]. However, the existence of a global liquid water layer on Triton has not yet been confirmed due to limited flyby data [62]. Consequently, NASA has proposed the Trident Discovery-class mission concept [92] and the Neptune Odyssey flagship concept [93] to resolve these fundamental questions.

Cross-disciplinary studies of small bodies and icy satellites reveal that prebiotic materials exhibit a distributed evolutionary pattern across the Solar System. Although small bodies and icy satellites differ greatly in properties and research methods, they jointly form key links in unraveling the chain of life’s origin. Prebiotic organic matter has undergone a distributed process from initial synthesis, through cross-scale transport, to multi-environment transformation and continuous evolution during the formation and evolution of the Solar System: carbonaceous small bodies serve as important sources and carriers of organic matter, delivering complex organic molecules to terrestrial planets (e.g., Mars), while icy satellites, through their long-term stable subsurface oceans and water–rock interaction environments, provide critical conditions for the sustained evolution and complexification of organic compounds. This process delineates the origin, distribution, and environmentally driven evolution of organic matter on a Solar System scale, providing key scientific foundations for constructing a cross-scale evolutionary framework from prebiotic chemistry to the origin of life.

### 2.2 Advance in deep space exploration missions

Building upon its extensive exploration of the major planets, their satellites, and small bodies within the Solar System, the United States has recently identified the search for extraterrestrial biosignatures and the study of planetary habitability as its highest-priority scientific objectives. The *Planetary Science and Astrobiology Decadal Survey 2023–2032* emphasizes investigating the evolution of planetary habitability and the co-evolution of planets and life [81]. As summarized in Table 1 and Table 2, five of the eight Flagship missions currently implemented or under development, along with three of the four New Frontiers missions, are directly related to this theme.

**Table 1:** **NASA Flagship missions**.

**Table 2:** **NASA New Frontiers missions**.

Among these, the Mars Sample Return (MSR) mission—designated as NASA’s sixth Flagship mission and the top priority during the 2013–2022 planning cycle—aims to collect rock and dust samples from Mars and return them to Earth. The mission is designed to search for potential signs of ancient life in the samples and advance understanding of Martian geological processes and environmental history. However, due to severe cost overruns, the U.S. Congress officially canceled funding for the mission in January 2026, leaving the samples already collected by Perseverance unable to be returned to Earth. Nevertheless, the Perseverance rover continues to collect Martian samples. The Europa Clipper mission, NASA’s seventh Flagship mission launched on October 14, 2024, will conduct multiple flybys of Jupiter’s satellite Europa from orbit to perform detailed observations of its ice shell, potential subsurface ocean, surface chemistry, and geological activity, assessing its habitability and searching for indications of life. The Uranus Orbiter and Probe (UOP), planned as NASA’s eighth Flagship mission for launch in the 2030s, is designed to investigate Uranus’s formation and orbital migration history, study the planet’s internal structure, atmosphere, magnetosphere, and ring system, and examine whether its icy satellites may harbor liquid water oceans with potential habitability.

Building upon its long-term scientific roadmap, the European Space Agency has established the search for extraterrestrial biosignatures and the study of planetary habitability as central objectives of its future exploration agenda. The *Voyage 2050 Long-term Planning of the ESA Science Program White Papers* (COSMOS White Papers - Voyage 2050 - Cosmos), which guides the agency’s priorities through 2050, outlines several key mission themes dedicated to this pursuit. The Jupiter Icy Moons Explorer (JUICE) mission, launched in 2023, is currently operational and aims to conduct detailed investigations of the subsurface oceans of Ganymede, Callisto, and Europa to assess their habitability and search for potential biosignatures. Looking ahead, the planned Large-class mission L4 is designed to further explore the icy satellites of giant planets, representing a direct extension and enhancement of the JUICE mission’s objectives. Furthermore, the Large-class mission L6 will focus on characterizing “temperate” exoplanets via direct imaging techniques, thereby expanding the search for life beyond our Solar System. Complementing these Large-class missions, a dedicated Medium-class mission targeting the exploration of ice giants or Neptune-like planets has also been incorporated into the portfolio. Collectively, these missions form a cohesive and systematic strategic framework for the search for life, spanning from the inner to the outer Solar System and extending into the galactic realm.

China has also placed high priority on the search for extraterrestrial biosignatures, incorporating it into national-level strategic plans and mission designs. In September 2020, the Central Government approved the Planetary Exploration Program as a National Major Project, with the Tianwen-3 mission designating the search for biosignatures on Mars as its primary scientific objective [94]. Furthermore, China’s recently released *National Space Science Mid- and Long-Term Development Plan (2024–2050)* (National Mid- and Long-term Plan for Space Science in China (2024–2050) —-National Space Science Center, CAS) explicitly identified “the search for extraterrestrial biosignatures and the study of planetary habitability” as a prioritized direction for space science development in China, calling for further demonstration and implementation of dedicated space science missions in this domain.

## 3 Key scientific themes

Current biosignature detection strategies are increasingly focused on potentially habitable niches such as shallow subsurface fluid systems and subglacial oceans, where several promising findings have already been made. A critical pathway to understanding the origin and evolution of life lies in investigating the transition from prebiotic worlds to living systems and establishing the co-evolutionary relationships between life’s origin, propagation, and multi-sphere coupling within habitable environments.

Building on extensive prior exploration and numerous significant discoveries, the search for extraterrestrial biosignatures is now at a pivotal stage—transitioning from incremental accumulation to qualitative breakthroughs and from fragmented efforts to systematic investigation. Through well-structured and system-wide research initiatives in the detection and study of extraterrestrial biosignatures, groundbreaking scientific discoveries are anticipated, which could ultimately lead to transformative advances in human civilization.

To systematically establish the scientific foundation and methodological framework for extraterrestrial biosignature detection, this study centers on the core conceptual chain of “planetary environmental habitability–presence of biosignatures–co-evolution of life and environment” to identify three interrelated scientific themes, under which eight key scientific questions are formulated. These themes advance progressively across three dimensions—environmental conditions, evidence of life, and interaction mechanisms: Theme I focuses on the formation and maintenance mechanisms of habitable environments; Theme II addresses the detection and identification of biosignatures; and Theme III elucidates the laws of life-environment interactions. Below, the three scientific themes and their associated key scientific questions are detailed.

### 3.1 Scientific theme I: planetary habitability

(1) Formation and evolution of planetary habitable environments

This research aims to systematically reveal the formation and evolution mechanisms of planetary habitability. It investigates the origin and enrichment mechanisms of water and volatiles during early planetary formation, examines the contribution of primitive materials from asteroids and comets to early Solar System habitability, and analyzes how internal structure, thermal evolution, and geological processes (e.g., plate tectonics, volcanism) regulate environmental stability. This research also addresses atmospheric escape, redox evolution, and climate feedback mechanisms, and uses Venus as a key case to explore factors driving the transition from habitable conditions to a runaway greenhouse state. By developing models of material transport chains and inversion methods for evolutionary pathways, this research seeks to reveal universal features and divergences in planetary habitability, providing a theoretical framework for assessing the habitability of exoplanets.

(2) Types of celestial bodies and possible scenarios for habitable environments

This research is dedicated to systematically identifying environmental characteristics of celestial bodies capable of supporting life, with emphasis on the formation, evolution, and multi-layer structural features of various bodies within the Solar System. It analyzes material and energy transport mechanisms in planetary systems to reveal how habitability manifests under different orbital positions, atmospheric compositions, geological activities, and magnetic shielding conditions. The scope covers habitability assessments from traditional terrestrial planets to unique environments (e.g., local ice-rock mixtures or cavity microenvironments in asteroids and comets). Models such as “cold-source seeding” are used to analyze the role of cometary impacts in promoting early habitable environments. The research aims to systematically construct possible scenarios and criteria for identifying habitable settings, offering theoretical foundations and observational constraints for exoplanet habitability studies.

(3) Commonalities and differences in planetary habitable environments

Through a multidimensional comparative analytical framework, this research systematically investigates key parameters—such as orbital properties, climatic conditions, atmospheric composition, and surface environments—across various bodies in the Solar System. Key focuses include: analyzing how stellar type and orbital dynamics regulate long-term planetary habitability; revealing contributions of various energy sources (solar radiation, tidal heating, radioactive decay, hydrothermal activity) to the formation and maintenance of habitable environments; comparing the occurrence states and sustaining conditions of liquid water on different bodies; developing coupled climate-chemical environment models and analyzing their variations across celestial bodies; and exploring the relationship between the distribution of organic materials in asteroids and comets and planetary habitability. Technologically, emphasis is placed on developing specialized extreme-environment protection systems, creating embedded platforms for climate simulation and environmental feedback modeling, and advancing in-situ detection and remote sensing technologies suited for multi-body comparative studies, thereby providing a robust scientific foundation and comprehensive technical support for future planetary habitability assessment.

### 3.2 Scientific theme II: search for extraterrestrial biosignatures

(1) Existence of living organisms

This research aims to explore the possibility and forms of extraterrestrial life, focusing on analyzing basic characteristics and conditions for potential life; assessing the potential for non-carbon-based life in extreme settings (e.g., subsurface oceans of icy satellites); studying adaptation mechanisms and survival limits of life in extreme environments; and investigating possible biological residues, cellular structures, or membranous features preserved in comets and asteroids. Through developing planetary-scale habitability models and probabilistic inference methods integrated with astrochemical and geological evolution frameworks, it systematically evaluates the potential for the origin of life on Solar System bodies. Technologically, priority is given to developing high-sensitivity in-situ biosignature detection techniques, including mass spectrometers, Raman spectrometers, DNA selectors, and in-situ microscopic imaging systems with micron-to nano-scale resolution, providing scientific methods and technical support for extraterrestrial biosignature detection.

(2) Biosignatures and biomarkers

This research focuses on characterizing biosignatures and distinguishing them from abiotic features, including: systematically studying the types and distribution patterns of biomarkers (e.g., specific molecules, isotopic anomalies, biogenic structures) in the Solar System; discriminating between biogenic and abiogenic origins of potential biosignatures and establishing effective identification criteria; investigating the forms, preservation media, and high-probability preservation zones of biosignatures in different celestial environments; and developing integrated discrimination methods combining multi-source evidence (morphological, compositional, spectral). Technologically, the research emphasizes in-situ biomolecular detection equipment, high-precision isotope analyzers, gas composition analysis systems (for detecting biogenic gases like CH_4_, O_2_, N_2_O), non-equilibrium state discriminators, as well as sample encapsulation and contamination prevention systems, offering methodological and technical support for accurate identification and verification of extraterrestrial biosignatures.

(3) Approaches for detecting and identifying biosignatures

This research focuses on developing novel methods for capturing and identifying extremely weak biosignatures, including: systematically studying detection methods and environmental characteristics across different extraterrestrial settings to formulate targeted biosignature search strategies; advancing signal enhancement and extraction techniques for faint biosignatures and overcoming challenges in low signal-to-noise scenarios; refining atmospheric spectral inversion algorithms for exoplanets to improve recognition accuracy of biosignature spectral features; and designing scientific landing and drilling sampling strategies covering shallow/deep layers and in-situ/return modes. Key technology development includes: integrated landing and drilling systems for planetary surfaces; machine learning-based intelligent big data analysis algorithms; Venus high-altitude floating platform technology; multi-point sampling and high-precision analysis techniques for comets/asteroids; and laboratory-based sealing and preservation techniques for returned samples from Mars, comets, asteroids, and other bodies, as well as high-precision detection and analysis methods for potential organic compounds and microorganisms within these samples, providing advanced technical means and methodological support for extraterrestrial biosignature detection.

### 3.3 Scientific theme III: co-evolution of life and habitable environments

(1) How habitable environments trigger the origin of life

This research examines environmental triggering mechanisms for the origin of life. Research focuses include: investigating environmental elements, material bases, and physicochemical conditions required for life’s origin; analyzing effects of spatial stress environments on genomic stability; studying evolutionary pathways from disorder to order and adaptation strategies to extreme environments; revealing transformation and polymerization potential of cometary and asteroidal organic materials in terrestrial-planet hydrothermal settings; simulating roles of early Venusian liquid water environments in chemical pathways toward life; and validating driving mechanisms such as impact events, water-rock reactions, and tidal heating in celestial environments. Technologically, emphasis is on constructing multi-parameter-controlled primitive environment simulation systems; developing instruments to monitor molecular assembly and self-organization reactions; and creating in-situ primitive chemical reaction analysis platforms with microfluidic systems, providing experimental support and technical means for studying environmental boundary conditions of life’s origin.

(2) Transformation of habitable environments by Life

This research investigates interaction mechanisms between potential extraterrestrial biological activities and environmental evolution, including: analyzing possible utilization of atmospheric components (e.g., methane metabolism, sulfur cycling) by extraterrestrial life and the resulting metabolic products; revealing biogeochemical cycles (carbon, nitrogen, sulfur, etc.) and environmental feedback mechanisms involving biology; establishing post-origin atmospheric evolution models (e.g., oxygen accumulation processes); studying influences of microbial communities on geological processes such as mineral deposition and climate regulation; and conducting interplanetary comparative studies on biologically driven environmental evolution to assess the long-term maintenance of habitability and ecosystem stability under biological influence. Technologically, priorities include developing planetary-scale atmospheric evolution monitoring equipment, deploying surface and subsurface sensor networks for long-term chemical cycle monitoring, and constructing bio-environment feedback numerical models and simulation platforms, providing observational tools and research platforms for uncovering principles of life-environment co-evolution.

## 4 Research targets and detection strategies

Building on the systematic review of research progress and the synthesis of the three key scientific themes outlined above, this section identifies priority target celestial bodies for future research on extraterrestrial biosignatures and planetary habitability and argues for key detection scenarios. The selection criteria are closely aligned with the scientific themes, focusing on those bodies and environments that hold the greatest potential for addressing the core questions of “planetary habitability”, “presence of biosignatures”, and “life-environment co-evolution”.

### 4.1 Target celestial bodies

Celestial bodies within the Solar System that are potentially habitable or preserve critical evidence related to life primarily include planets (Mars, Venus), satellites (icy satellites), and small bodies (asteroids, comets). Their selection is based on whether they possess or once possessed fundamental elements of habitability (e.g., liquid water, suitable temperatures, energy sources, key organic molecules, etc.) and whether their scientific return can directly address the key scientific questions raised in Section 3.

Mars serves as a “natural laboratory” for studying the long-term evolution of planetary habitability and the preservation mechanisms of biosignatures. Its core scientific value lies in three aspects. First, the absence of global plate tectonics has preserved geological records from 3 to 4 billion years ago, providing a unique archive for studying the formation, persistence, and loss of habitable environments (addressing Question 1 under Scientific Theme I). Second, the discovery of mid-to-low latitude paleo-shallow marine sedimentary layers by the Zhurong rover [25], along with evidence of water-rock interactions and organic molecules found by the Perseverance rover in Jezero Crater [95], collectively extend Mars’ habitable window to hundreds of millions of years and point to potential habitats (addressing Questions 4 and 5 under Scientific Theme II). Third, its global climatic transition from warm and wet to cold and dry offers an extreme case for studying the co-evolution of life (if it ever existed) and the environment (addressing Question 8 under Scientific Theme III). Exploring Mars aims to quantify the duration of habitable environments, uncover mechanisms behind the termination of its water cycle, and establish standards for detecting extraterrestrial biosignatures.

China’s Tianwen-3 mission [94], scheduled for launch in 2028 and designed to return no <500 g of Martian samples to Earth by 2031, has core scientific objectives that align closely with the three themes above. Regarding planetary habitability, the mission will systematically reconstruct the evolutionary history of Martian aqueous environments by combining data from the lander-borne subsurface penetrating radar and the orbiter-based mid-infrared hyperspectral imager with multi-isotope and volatile analyses of the returned samples (addressing Question 1 under Scientific Theme I). For the search for biosignatures, Tianwen-3 adopts a full-chain investigation strategy—from landing-site selection and sample collection (surface scooping, 2 m drilling, and helicopter sampling) to analysis using Raman-fluorescence spectrometry and high-precision isotope measurements in the Mars Sample Laboratory—enabling the discrimination between biogenic and abiogenic organic molecules and isotopic anomalies (addressing Questions 4, 5, and 6 under Scientific Theme II). On life-environment co-evolution, the mission will analyze early water-rock interactions, methane fluctuations, and volcanic records on Mars, thereby revealing the mechanisms that triggered and terminated habitable environments (addressing Questions 7 and 8 under Scientific Theme III). As the first Mars sample-return mission implemented under the highest planetary protection category (COSPAR Category V, restricted return), Tianwen-3, with its integrated “in situ detection – sample return – dedicated laboratory” chain, will provide direct evidence for the search for biosignatures and the evolution of habitability on Mars.

Venus provides a comparative model for studying critical transitions in planetary habitability and the potential for life in non-aqueous solvents. Its scientific uniqueness stems from three aspects. First, as Earth’s “sister planet”, its evolution into an extreme greenhouse world (surface temperature 462 °C) is key to understanding the mechanisms of habitability loss on terrestrial planets (addressing Question 1 under Scientific Theme I). Second, the temperate pressure and temperature conditions in its upper cloud layer (50–65 km) [38], detected phosphine signals [42], and theoretical stability of nucleic acid bases in sulfuric acid [96] together make it a frontier for exploring non-water-based life (sulfuric acid solvent) and its biomarkers (addressing Questions 4 and 5 under Scientific Theme II). Exploring Venus will help develop a theory of planetary climate “tipping points”, test the limits of life in extreme environments, and significantly expand the definition of habitability.

Icy satellites (represented by Europa, Enceladus, and Triton) are potential targets for exploring the origin of life and independent life systems in the Solar System [97]. Their core value lies in three aspects. First, they possess global subsurface oceans, maintained by tidal heating, providing long-term stable liquid water environments and energy sources—exemplifying “long-term stable habitability” (addressing Question 2 under Scientific Theme I). Second, Cassini confirmed that Enceladus’ plumes contain key life-related components such as phosphorus and organic compounds, demonstrating a complete chemical chain capable of supporting life (e.g., chemosynthesis) [78,[98], [99], [100]] (addressing Question 4 under Scientific Theme II). Third, their enclosed ocean-rock systems provide perfect models for studying how life could originate and evolve independently in environments starkly different from Earth’s (addressing Question 7 under Scientific Theme III). Among these, Triton—possibly a captured Kuiper Belt object [101]—may contain more primitive organic material, offering a new perspective on habitability and the origin of life in non-traditional environments, such as ammonia-water mixed solvents (addressing Scientific Themes I and III). Exploring icy satellites aims to verify low-temperature metabolic pathways and explore forms of life in completely isolated systems.

Small bodies (carbonaceous asteroids and comets) are “time capsules” for tracing the sources of life’s precursor materials and the early chemical evolution of the Solar System. Their irreplaceable role includes three aspects. First, they preserve pristine material from the solar nebula 4.6 billion years ago, such as the abundant organic matter discovered by the Hayabusa2 and Rosetta missions [65,102], serving as starting points for studying the material basis of habitability (addressing Question 3 under Scientific Theme I). Second, the composition, distribution, and abundance of their organic matter provide direct samples for understanding the cosmic distribution of prebiotic chemistry (addressing Question 5 under Scientific Theme II). Third, they may have delivered life’s building blocks to inner planets through impacts, directly participating in the construction of habitable environments and the origin of life on terrestrial planets (addressing Question 7 under Scientific Theme III). Exploring small bodies aims to reveal the earliest material evolution in the Solar System and provide key evidence for the “exogenous delivery” hypothesis of life’s origin.

In summary, selecting Mars, Venus, icy satellites (e.g., Europa, Enceladus, Triton), and small bodies as priority targets forms a well-structured, science-driven target system: Venus explores the limits of habitability loss, Mars examines the duration of habitability, icy satellites probe the potential for independent origin of life in isolated systems, and small bodies trace the material source of all these processes.

### 4.2 Detection strategies

Based on the scientific understanding of the above target bodies, future exploration must focus on specific scenarios most likely to preserve evidence of habitable environments or biosignatures. Their selection directly serves the methods for biosignature detection and identification (Scientific Theme II).

#### 4.2.1 Mars: paleo-aqueous environments and shallow subsurface

Detection will focus on (a) Paleo-lake/ocean sedimentary sequences (e.g., Jezero Crater, Utopia Planitia), drilling to obtain cores for analyzing sedimentary structures, mineralogy (e.g., clays, carbonates), and organic molecules, aiming to discover microbial fossils or biogenic mineral evidence; (b) modern shallow subsurface brine regions and groundwater ice, using penetrating radar to locate and sample at depth, detecting extant halophilic microorganisms or their biomarkers; and (c) modern methane release source regions, conducting long-term in-situ monitoring to distinguish between biological and abiotic origins. These scenarios are central to answering “whether life traces exist” and “how they are preserved”.

#### 4.2.2 Venus: cloud layer in the upper atmosphere

The detection scenario is highly concentrated in the cloud layer 50–65 km above the surface [30]. Future missions will deploy floating platforms or aircraft to traverse this region, directly collecting cloud droplet aerosol samples to analyze their chemical composition (especially phosphine, sulfuric acid, complex organics), particle morphology, and possible microbial structures. This is the only scenario for testing the “non-water-based life” hypothesis and exploring the diversity of life forms.

#### 4.2.3 Icy satellites: subsurface oceans and their plumes

Key detection scenarios include (a) plume venting: performing multiple fly-throughs and sampling of plume materials from bodies like Enceladus and Europa to comprehensively analyze water chemistry, complexity of organic macromolecules, chiral characteristics, and abundance of key elements such as phosphates, directly assessing their potential to harbor life; (b) beneath the ice shell/ice-water interface: developing penetration technologies such as melting probes to reach the subsurface ocean, detecting its salinity, pH, redox state, and potential hydrothermal vent systems, searching for ecosystems analogous to Earth’s black smokers. This is the optimal scenario for exploring “independent life systems” and “an origin of life”.

#### 4.2.4 Small bodies: pristine samples and dynamic outgassing

Detection scenarios include (a) asteroid/comet surface and subsurface: Through sample return (e.g., Tianwen-2) or in-situ analysis, obtaining pristine material minimally affected by space weathering, precisely determining types of organic molecules, isotopic composition, and amino acid enantiomeric excess; (b) active cometary outbursts: conducting fly-by observations of dynamic comets to capture released volatiles and dust, studying the evolution of organics under solar wind irradiation. These scenarios are key to revealing the “sources of life’s precursor materials” and “prebiotic chemical evolution”.

By prioritizing these high-value scenarios, limited exploration resources can be most effectively concentrated on directions with the greatest potential for major scientific breakthroughs, systematically addressing the core proposition of planetary habitability evolution and the search for extraterrestrial biosignatures.

## 5 Research foundation and development outlook

### 5.1 Research foundation

In terms of fundamental scientific research, the major planetary exploration program approved by the Chinese central government places significant emphasis on the search for biosignatures and the study of planetary habitability. The Tianwen-1 mission has been successfully implemented, yielding new evidence of aeolian and aqueous activities from the Late Amazonian period (approximately 3 billion years ago) through studies of Martian dune and mud volcano landforms, soil physical properties, mineral and rock compositions, and shallow subsurface structures. The Tianwen-3 mission is currently in the design phase and will focus on investigating potential biosignatures, Martian climate and its evolution, as well as geological and internal processes.

Regarding key technological development, institutions such as the State Administration for Science, Technology and Industry for National Defense (SASTIND) and the National Natural Science Foundation of China (NSFC) have allocated hundreds of millions of yuan in funding to support strategic research on the detection of extraterrestrial biosignatures and the evolution of habitability, as well as mission concept design. These efforts aim to advance key technologies including the detection of habitable planets and biosignatures, atmospheric lift-based sampling platforms for extraterrestrial bodies, and small, highly reliable, long-lived space nuclear power systems. Additionally, scientific studies have been supported in areas such as Mars landing site selection, metal layers in the middle and upper atmosphere, and changes in the near-Earth space environment.

### 5.2 Development outlook

Research on extraterrestrial biosignatures and planetary habitability requires the establishment of a coordinated scientific exploration system targeting multiple celestial bodies. It is recommended to prioritize breakthroughs in three major directions: investigating the relationship between atmospheric chemical processes and potential biosignatures in Venusian clouds, with a focus on sulfur cycle mechanisms and the origin of ultraviolet absorbers; studying the preservation mechanisms of biosignatures on Mars to reveal the influence of geological processes on biosignatures; and assessing the habitability of subsurface oceans on icy satellites by exploring the preservation and transport mechanisms of key biomolecules within ice-water-rock systems. Major efforts should be devoted to constructing ground-based planetary environment simulation facilities capable of replicating the unique environmental conditions of multiple celestial bodies, thereby providing experimental support for research on environmental boundaries for the origin of life and the evolution of habitability. Through interdisciplinary research, criteria for biomarker identification and procedures for avoiding false positives should be established to deepen scientific understanding of extraterrestrial life forms and detection methods.

#### 5.2.1 Development outlook for Venus habitability research

Future research on Venusian habitability will focus on multi-sphere coupling and the detection of biosignatures, emphasizing atmospheric escape mechanisms and evolutionary history, the relationships among the atmosphere, the magnetic field, and the water cycle, the coupling between surface geological processes and atmospheric energy exchange, as well as studies of sulfur cycle mechanisms and isotopic composition in the clouds. Concurrently, efforts will be dedicated to unveiling the origin of ultraviolet absorbers in the clouds and their potential connection to biological activity, developing molecular-scale models of photophysical and photochemical transformations. This research aims to address key scientific questions such as the history of atmospheric escape and its correlation with potential cloud-based biosignatures, striving to deepen theoretical understanding of planetary habitability evolution and extraterrestrial biosignature detection through interdisciplinary approaches and methodological innovation.

#### 5.2.2 Development outlook for the search for biosignatures on Mars

Research on Martian biosignatures will center on environmental mechanisms of the origin and preservation of life, as well as innovations in detection methodologies. Core scientific issues include atmospheric escape and habitability evolution, distribution patterns of water ice, multi-sphere coupling processes, and mechanisms of life-environment co-evolution. Key focus will be placed on investigating the occurrence patterns of biosignatures within carriers such as clay minerals and saline deposits, and their alteration by geological processes including aqueous activity and impact events. Relying on terrestrial analog environments in extreme settings, studies will analyze microbial community structures, metabolic features, and biomarkers in arid and high-radiation environments to infer survival strategies and preservation modes of potential life on Mars. By constructing ground-based Martian environment simulation facilities, environmental evolution processes will be replicated to reveal thresholds for life’s origin and extinction, and to assess the transformative effects of geological events on the biosphere. The research aims to develop highly sensitive detection methods, establish evaluation models for biogenic minerals and gases, and enhance understanding of Martian habitability evolution and the distribution patterns of biosignatures.

#### 5.2.3 Development outlook for the search for biosignatures in the subsurface oceans of icy satellites

Life detection on icy satellites will focus on assessing the habitability of subsurface oceans and identifying potential biosignatures. Core scientific questions include the interaction mechanisms between icy satellites and their host planets, cross-sphere material and energy transport, evolution of ocean chemical composition, and spatiotemporal boundaries for the origin of life. Emphasis will be placed on exploring traces of prebiotic chemical evolution, life forms in extreme environments, and the preservation and transport patterns of their metabolic products within ice-water-rock systems. Future research should develop planetary spectroscopy analysis systems, infer ocean characteristics through induced magnetic field measurements, and enhance laboratory simulations of icy satellite environments to validate the stability and detection thresholds of key biomolecules. Theoretically, chemical evolution models should be employed to define environmental boundaries for life’s origin, establish identification criteria and false-positive exclusion procedures for biomarkers (e.g., chiral molecules, isotopic fractionation), and deepen research on the relationship between ocean evolution and life potential, thereby providing a scientific basis for the detection of extraterrestrial biosignatures.

## 6 Summary and outlook

The search for extraterrestrial biosignatures and the study of planetary habitability represent a major frontier in understanding the origin of life and its potential existence beyond Earth. This paper has systematically elaborated on three core themes in this field: the formation and evolution of planetary habitability, the detection and identification of biosignatures, and the co-evolution of life and environment, proposing a multi-target collaborative research framework centered on Venus, Mars, and icy satellites. Venus offers a unique case for studying critical transition mechanisms in the habitability of terrestrial planets; Mars serves as a key target for investigating the duration of habitable environments and the preservation patterns of biosignatures; while icy satellites expand the physicochemical boundaries of potential life. This study emphasizes the necessity of constructing large-scale ground-based planetary simulation facilities to replicate extreme multi-body environments and overcome technical bottlenecks in biosignature detection and interpretation.

Future research should foster deeper interdisciplinary integration, combining methods from geology, astrochemistry, and biology to systematically reveal multi-sphere coupling mechanisms on planets and the environmental constraints on life’s origin. Priority should be given to developing highly sensitive detection technologies, establishing criteria for biomarker identification based on multi-source information fusion, and enhancing the scientific return of deep space exploration data. Through systematic research on Venus, Mars, and icy satellites, breakthroughs are anticipated in fundamental scientific areas such as the origin of life and the formation of the Solar System, shedding light on the evolution of planetary habitable environments and the material and energy foundations of life.

In the long term, the search for extraterrestrial biosignatures will not only address the fundamental question of the distribution of life in the universe but also deepen our understanding of the principles of life’s origin and evolution. China should continue to strengthen basic research in this field, cultivate high-level scientific teams, build indigenous innovation capabilities, and play a significant role in international planetary science.

## CRediT authorship contribution statement

**Zengqian Hou:** Writing – review & editing, Writing – original draft, Validation, Supervision, Resources, Methodology, Investigation, Funding acquisition, Formal analysis, Conceptualization. **Jizhong Liu:** Writing – review & editing, Writing – original draft, Validation, Supervision, Methodology, Investigation, Conceptualization. **Yuming Wang:** Writing – review & editing, Writing – original draft, Validation, Supervision, Resources, Methodology, Investigation, Funding acquisition, Conceptualization. **Lei Geng:** Writing – review & editing, Writing – original draft, Validation, Supervision, Resources, Methodology, Investigation, Funding acquisition, Conceptualization. **Hui Huang:** Writing – review & editing, Writing – original draft, Validation, Investigation. **Hailiang Dong:** Writing – review & editing, Writing – original draft, Validation, Investigation. **Yazhou Dong:** Writing – review & editing, Writing – original draft, Validation, Investigation. **Siteng Fan:** Writing – review & editing, Writing – original draft, Validation, Investigation. **Jihua Hao:** Writing – review & editing, Writing – original draft, Validation, Investigation. **Jiansen He:** Writing – review & editing, Writing – original draft, Validation, Investigation. **Dali Kong:** Writing – review & editing, Writing – original draft, Validation, Investigation. **Hui Li:** Writing – review & editing, Writing – original draft, Validation, Investigation. **Yan Li:** Writing – review & editing, Writing – original draft, Validation, Investigation. **Yiliang Li:** Writing – review & editing, Writing – original draft, Validation, Investigation. **Mang Lin:** Writing – review & editing, Writing – original draft, Validation, Investigation. **Zongcheng Ling:** Writing – review & editing, Writing – original draft, Validation, Investigation. **Yuangao Qu:** Writing – review & editing, Writing – original draft, Validation, Investigation. **Zhenfeng Sheng:** Writing – review & editing, Writing – original draft, Validation, Investigation. **Tong Wang:** Writing – review & editing, Writing – original draft, Validation, Investigation. **Yuxian Wang:** Writing – review & editing, Writing – original draft, Validation, Investigation. **Bo Xu:** Writing – review & editing, Writing – original draft, Validation, Investigation. **Jun Yang:** Writing – review & editing, Writing – original draft, Validation, Investigation. **Bingkun Yu:** Writing – review & editing, Writing – original draft, Validation, Investigation, Funding acquisition. **Xiao Zhang:** Writing – review & editing, Writing – original draft, Validation, Investigation.

## Declaration of competing interest

Zengqian Hou, Yuming Wang and Bingkun Yu are Guest Editors for this journal and were not involved in the editorial review or the decision to publish this article. The authors declare that they have no conflicts of interest in this work.

## References

[1] Martin W., Russell M.J. (2003). On the origins of cells: A hypothesis for the evolutionary transitions from abiotic geochemistry to chemoautotrophic prokaryotes, and from prokaryotes to nucleated cells. Philos. Trans. R. Soc. Lond. B: Biol. Sci..

[2] Arrhenius S. (1908). Worlds in the making: The evolution of the universe. Harper.

[3] Arndt N.T., Nisbet E.G. (2012). Processes on the young Earth and the habitats of early life. Annu. Rev. Earth Planet. Sci..

[4] Trail D., Watson E.B., Tailby N.D. (2011). The oxidation state of hadean magmas and implications for early Earth’s atmosphere. Nature.

[5] Wordsworth R.D. (2016). The climate of early Mars. Annu. Rev. Earth Planet. Sci..

[6] Elmasry W., Kebukawa Y., Kaneko T. (2020). Alteration and stability of complex macromolecular amino acid precursors in hydrothermal environments. Orig. Life Evol. Biosph..

[7] Martin W., Baross J., Kelley D. (2008). Hydrothermal vents and the origin of life. Nat. Rev. Microbiol..

[8] Pizzarello S., Williams L.B., Lehman J. (2011). Abundant ammonia in primitive asteroids and the case for a possible exobiology. Proc. Natl. Acad. Sci..

[9] Russell M.J., Hall A.J., Martin W. (2010). Serpentinization as a source of energy at the origin of life. Geobiology.

[10] Zahnle K.J., Lupu R., Catling D.C. (2020). Creation and evolution of impact-generated reduced atmospheres of early Earth. Planet. Sci. J..

[11] Kobayashi K., Ise J.-I., Aoki R. (2023). Formation of amino acids and carboxylic acids in weakly reducing planetary atmospheres by solar energetic particles from the young sun. Life.

[12] Beyazay T., Belthle K.S., Farès C. (2023). Ambient temperature CO2 fixation to pyruvate and subsequently to citramalate over iron and nickel nanoparticles. Nat. Commun..

[13] Ertem G., Ferris J.P. (1996). Synthesis of RNA oligomers on heterogeneous templates. Nature.

[14] Hazen R.M., Sverjensky D.A. (2010). Mineral surfaces, geochemical complexities, and the origins of life. Cold Spring Harb. Perspect. Biol..

[15] Mizuuchi R., Ichihashi N. (2018). Sustainable replication and coevolution of cooperative RNAs in an artificial cell-like system. Nat. Ecol. Evol..

[16] Aithal A., Dagar S., Rajamani S. (2023). Metals in prebiotic catalysis: A possible evolutionary pathway for the emergence of metalloproteins. ACS Omega.

[17] Jordan S.F., Ioannou I., Rammu H. (2021). Spontaneous assembly of redox-active iron-sulfur clusters at low concentrations of cysteine. Nat. Commun..

[18] Charnay B., Forget F., Wordsworth R. (2013). Exploring the faint young Sun problem and the possible climates of the Archean Earth with a 3-D GCM. J. Geophys. Res.: Atmos..

[19] Timmermann A., Yun K.-S., Raia P. (2022). Climate effects on archaic human habitats and species successions. Nature.

[20] Wang Y., Liu J., Liu L. (2026). Formation, evolution, and habitability of terrestrial planets: A review of research progress. Sci. China Earth Sci..

[21] Lucchitta B.K., Ferguson H., Summers C. (1986). Sedimentary deposits in the northern lowland plains, Mars. J. Geophys. Res.: Solid Earth.

[22] Parker T.J., Gorsline D.S., Saunders R.S. (1993). Coastal geomorphology of the Martian northern plains. J. Geophys. Res.: Planets.

[23] Kreslavsky M.A., Head J.W. (2002). Fate of outflow channel effluents in the northern lowlands of Mars: The Vastitas Borealis Formation as a sublimation residue from frozen ponded bodies of water. J. Geophys. Res.: Planets.

[24] Ivanov M., Erkeling G., Hiesinger H. (2017). Topography of the Deuteronilus contact on Mars: Evidence for an ancient water/mud ocean and long-wavelength topographic readjustments. Planet Space Sci..

[25] Li J., Liu H., Meng X. (2025). Ancient ocean coastal deposits imaged on Mars. Proc. Natl. Acad. Sci..

[26] Hou B., Sun H., Hu Z. (2026). Manganese (Hydr) oxides record the dynamic evolution of a million-year Hesperian Ocean in Utopia Planitia, Mars. Nat. Commun..

[27] Léveillé R.J., Datta S. (2010). Lava tubes and basaltic caves as astrobiological targets on Earth and Mars: A review. Planet Space Sci..

[28] Sauro F., Pozzobon R., Massironi M. (2020). Lava tubes on Earth, Moon and Mars: A review on their size and morphology revealed by comparative planetology. Earth-Sci. Rev..

[29] Palma V., De la Rosa J.M., Onac B.P. (2024). Decoding organic compounds in lava tube sulfates to understand potential biomarkers in the Martian subsurface. Commun. Earth Environ..

[30] Wang Y., Yu B., Geng L. (2026). Scientific themes in Venus exploration. Fundam. Res..

[31] Mahieux A., Viscardy S., Yelle R.V. (2024). Unexpected increase of the deuterium to hydrogen ratio in the Venus mesosphere. Proc. Natl. Acad. Sci..

[32] Donahue T., Hoffman J., Hodges R. (1982). Venus was wet: A measurement of the ratio of deuterium to hydrogen. Science.

[33] McElroy M.B., Prather M.J., Rodriguez J.M. (1982). Escape of hydrogen from Venus. Science.

[34] Way M.J., Del Genio A.D. (2020). Venusian habitable climate scenarios: Modeling Venus through time and applications to slowly rotating Venus-like exoplanets. J. Geophys. Res.: Planets.

[35] Way M.J., Del Genio A.D., Kiang N.Y. (2016). Was Venus the first habitable world of our solar system?. Geophys. Res. Lett..

[36] Yang J., Cowan N.B., Abbot D.S. (2013). Stabilizing cloud feedback dramatically expands the habitable zone of tidally locked planets. Astrophys. J. Lett..

[37] Turbet M., Bolmont E., Chaverot G. (2021). Day–night cloud asymmetry prevents early oceans on Venus but not on Earth. Nature.

[38] Seager S., Petkowski J.J., Gao P. (2021). The Venusian lower atmosphere haze as a depot for desiccated microbial life: A proposed life cycle for persistence of the Venusian aerial biosphere. Astrobiology.

[39] Limaye S.S., Grassi D., Mahieux A. (2018). Venus atmospheric thermal structure and radiative balance. Space Sci. Rev..

[40] Limaye S.S., Mogul R., Baines K.H. (2021). Venus, an astrobiology target. Astrobiology.

[41] J. Spacek, Organic carbon cycle in the atmosphere of Venus, arXiv preprint arXiv:2108.02286 (2021). 10.48550/arXiv.2108.02286.

[42] Greaves J.S., Richards A.M., Bains W. (2021). Phosphine gas in the cloud decks of Venus. Nat. Astron..

[43] Akins A.B., Lincowski A.P., Meadows V.S. (2021). Complications in the ALMA detection of phosphine at Venus. Astrophys. J. Lett..

[44] Encrenaz T., Greathouse T.K., Marcq E. (2020). HDO and SO2 thermal mapping on Venus-V. Evidence for a long-term anti-correlation. Astron. Astrophys..

[45] Yin R., Li Y., Li Y. (2026). Phosphine depletion in brown dwarf atmospheres explained due to metal phosphide formation. Astrophys. J. Lett..

[46] Li Y., Yin R., Ye H. (2025). Photoactive elemental sulfur allotropes promote extensive ammonia synthesis in Venus-like atmosphere. Nat. Commun..

[47] Seager M.D., Seager S., Bains W. (2024). Stability of 20 biogenic amino acids in concentrated sulfuric acid: Implications for the habitability of Venus’ clouds. Astrobiology.

[48] Seager S., Petkowski J.J., Seager M.D. (2023). Stability of nucleic acid bases in concentrated sulfuric acid: Implications for the habitability of Venus’ clouds. Proc. Natl. Acad. Sci..

[49] Dundas C.M., Bramson A.M., Ojha L. (2018). Exposed subsurface ice sheets in the Martian mid-latitudes. Science.

[50] Holt J.W., Safaeinili A., Plaut J.J. (2008). Radar sounding evidence for buried glaciers in the southern mid-latitudes of Mars. Science.

[51] Orosei R., Lauro S.E., Pettinelli E. (2018). Radar evidence of subglacial liquid water on Mars. Science.

[52] Xu X., Xu Y., Han Z. (2025). Shallow water ice detection from SHARAD data in central utopia Planitia, Mars. J. Geophys. Res.: Planets.

[53] Li C., Zheng Y., Wang X. (2022). Layered subsurface in Utopia Basin of Mars revealed by Zhurong rover radar. Nature.

[54] Zhang L., Li C., Zhang J. (2024). Buried palaeo-polygonal terrain detected underneath Utopia Planitia on Mars by the Zhurong radar. Nat. Astron..

[55] Fedorova A.A., Montmessin F., Korablev O. (2020). Stormy water on Mars: The distribution and saturation of atmospheric water during the dusty season. Science.

[56] Carter J., Poulet F., Bibring J.P. (2013). Hydrous minerals on Mars as seen by the CRISM and OMEGA imaging spectrometers: Updated global view. J. Geophys. Res.: Planets.

[57] Bristow T.F., Rampe E.B., Achilles C.N. (2018). Clay mineral diversity and abundance in sedimentary rocks of Gale crater, Mars. Sci. Adv..

[58] Farley K., Stack K., Shuster D. (2022). Aqueously altered igneous rocks sampled on the floor of Jezero crater, Mars. Science.

[59] Liu Y., Wu X., Zhao Y.-Y.S. (2022). Zhurong reveals recent aqueous activities in Utopia Planitia, Mars. Sci. Adv..

[60] Qin X., Ren X., Wang X. (2023). Modern water at low latitudes on Mars: Potential evidence from dune surfaces. Sci. Adv..

[61] Hendrix A.R., Hurford T.A., Barge L.M. (2019). The NASA roadmap to ocean worlds. Astrobiology.

[62] Nimmo F., Pappalardo R.T. (2016). Ocean worlds in the outer solar system. J. Geophys. Res.: Planets.

[63] Brownlee D.E., Tsou P., Anderson J. (2003). Stardust: Comet and interstellar dust sample return mission. J. Geophys. Res.: Planets.

[64] Elsila J.E., Glavin D.P., Dworkin J.P. (2009). Cometary glycine detected in samples returned by Stardust. Meteorit. Planet Sci..

[65] Naraoka H., Takano Y., Dworkin J.P. (2023). Soluble organic molecules in samples of the carbonaceous asteroid (162173) Ryugu. Science.

[66] Lauretta D.S., Connolly H.C., Aebersold J.E. (2024). Asteroid (101955) Bennu in the laboratory: Properties of the sample collected by OSIRIS-REx. Meteorit. Planet Sci..

[67] Li C., Liu J., Ren X. (2024). Scientific objectives and payloads configuration of the Tianwen-2 mission. J. Deep Space Explor..

[68] Zhang R., Zhang H., Liu J. (2025). Tianwen-2 small bodies exploration mission. Sci. Sin. Phys. Mech. Astron..

[69] Castro-Cisneros J.D., Malhotra R., Rosengren A.J. (2023). Lunar ejecta origin of near-earth asteroid Kamo’oalewa is compatible with rare orbital pathways. Commun. Earth Environ..

[70] Jiao Y., Cheng B., Huang Y. (2024). Asteroid Kamo ‘oalewa’s journey from the lunar Giordano Bruno crater to Earth 1: 1 resonance. Nat. Astron..

[71] Sharkey B.N., Reddy V., Malhotra R. (2021). Lunar-like silicate material forms the Earth quasi-satellite (469219) 2016 HO3 Kamoʻoalewa. Commun. Earth Environ..

[72] Zhuge Z., Jiang T., Bian M. (2025). Design and calibration verification of the diffraction rotational snapshot spectral camera on the Tianwen-2 mission. Sci. Sin. Phys. Mech. Astron..

[73] Su B., Zhang A., Kong L. (2025). Charged and neutral particle analyzer for Tianwen-2 mission. Sci. Sin. Phys. Mech. Astron..

[74] De Mol M.L. (2023). Astrobiology in space: A comprehensive look at the solar system. Life.

[75] Dougherty M., Khurana K., Neubauer F. (2006). Identification of a dynamic atmosphere at Enceladus with the Cassini magnetometer. Science.

[76] Porco C.C., Helfenstein P., Thomas P. (2006). Cassini observes the active south pole of Enceladus. Science.

[77] Hansen C.J., Esposito L., Stewart A.I.F. (2006). Enceladus’ water vapor plume. Science.

[78] Postberg F., Khawaja N., Abel B. (2018). Macromolecular organic compounds from the depths of Enceladus. Nature.

[79] Waite J.H., Combi M.R., Ip W.-H. (2006). Cassini ion and neutral mass spectrometer: Enceladus plume composition and structure. Science.

[80] MacKenzie S.M., Neveu M., Davila A.F. (2021). The Enceladus Orbilander mission concept: Balancing return and resources in the search for life. Planet. Sci. J..

[81] National Academies of Sciences, Engineering, and Medicine (2023).

[82] Pappalardo R.T., Belton M.J., Breneman H. (1999). Does Europa have a subsurface ocean? Evaluation of the geological evidence. J. Geophys. Res.: Planets.

[83] Kivelson M.G., Khurana K.K., Russell C.T. (2000). Galileo magnetometer measurements: A stronger case for a subsurface ocean at Europa. Science.

[84] Jia X., Kivelson M.G., Khurana K.K. (2018). Evidence of a plume on Europa from Galileo magnetic and plasma wave signatures. Nat. Astron..

[85] Roth L., Saur J., Retherford K.D. (2014). Transient water vapor at Europa’s south pole. Science.

[86] Cangahuala L.A., Campagnola S., Bradley B.K. (2025). Europa clipper mission design, mission plan, and navigation. Space Sci. Rev..

[87] Grasset O., Dougherty M., Coustenis A. (2013). JUpiter ICy moons Explorer (JUICE): An ESA mission to orbit Ganymede and to characterise the Jupiter system. Planet Space Sci..

[88] Hansen C.J., Paige D.A. (1992). A thermal model for the seasonal nitrogen cycle on Triton. Icarus.

[89] Smith B.A., Soderblom L.A., Banfield D. (1989). Voyager 2 at Neptune: Imaging science results. Science.

[90] Moran S.E., Hörst S.M., He C. (2022). Triton haze analogs: The role of carbon monoxide in haze formation. J. Geophys. Res.: Planets.

[91] Pearce B.K., Hörst S.M., Cline C.J. (2024). Toward prebiotic chemistry on Titan: Impact experiments on organic haze particles. Planet. Sci. J..

[92] Sharma P., Lawler C.R., Mitchell K.L. (2022). 2022 IEEE Aerospace Conference (AERO).

[93] Rymer A.M., Runyon K.D., Clyde B. (2021). Neptune odyssey: A flagship concept for the exploration of the Neptune–Triton system. Planet. Sci. J..

[94] Hou Z., Liu J., Pang F. (2025). In search of signs of life on Mars with China’s sample return mission Tianwen-3. Nat. Astron..

[95] Sharma S., Roppel R.D., Murphy A.E. (2023). Diverse organic-mineral associations in Jezero crater, Mars. Nature.

[96] Seager S., Petkowski J.J., Seager M.D. (2024). Year-long stability of nucleic acid bases in concentrated sulfuric acid: Implications for the persistence of organic chemistry in Venus’ clouds. Life.

[97] Wang C., Li H., Wang Y. (2026). Triton as an archetype for icy Ocean worlds: Key scientific drivers for future exploration. Fundam. Res..

[98] Hao J., Glein C.R., Huang F. (2022). Abundant phosphorus expected for possible life in Enceladus’s ocean. Proc. Natl. Acad. Sci..

[99] Hsu H.-W., Schmidt J., Kempf S. (2018). In situ collection of dust grains falling from Saturn’s rings into its atmosphere. Science.

[100] Waite J.H., Glein C.R., Perryman R.S. (2017). Cassini finds molecular hydrogen in the Enceladus plume: Evidence for hydrothermal processes. Science.

[101] Goldreich P., Murray N., Longaretti P. (1989). Neptune’s story. Science.

[102] Goesmann F., Rosenbauer H., Bredehöft J.-H. (2015). The first mass spectrometry measurements of COSAC after touchdown on comet 67P/Churyumov-Gerasimenko. Science.

